# Data Mining Models in Prediction of Vancomycin-Intermediate *Staphylococcus aureus* in Methicillin-Resistant *S. aureus* (MRSA) Bacteremia Patients in a Clinical Care Setting

**DOI:** 10.3390/microorganisms13010101

**Published:** 2025-01-07

**Authors:** Wei-Chuan Chen, Jiun-Ling Wang, Chi-Chuan Chang, Yusen Eason Lin

**Affiliations:** 1 Division of Teaching and Education, Teaching and Research Department, Kaohsiung Veterans General Hospital, Kaohsiung 813414, Taiwan; wcchen1027@vghks.gov.tw (W.-C.C.);; 2 Department of Pharmacy and Master Program, Tajen University, Yanpu Township, Pingtung 907101, Taiwan; 3 Department of Nursing, Shu-Zen Junior College of Medicine and Management, Kaohsiung 821004, Taiwan; 4 Department of Internal Medicine, National Cheng Kung University Hospital, Tainan 701401, Taiwan; 5 Department of Medicine, College of Medicine, National Cheng Kung University, Tainan 701401, Taiwan; 6 Graduate Institute of Human Resource and Knowledge Management, National Kaohsiung Normal University, Kaohsiung 802561, Taiwan

**Keywords:** Vancomycin-intermediate *Staphylococcus aureus*, data mining techniques, risk factors

## Abstract

Vancomycin-intermediate *Staphylococcus aureus* (VISA) is a multi-drug-resistant pathogen of significant clinical concern. Various *S. aureus* strains can cause infections, from skin and soft tissue infections to life-threatening conditions such as bacteremia and pneumonia. VISA infections, particularly bacteremia, are associated with high mortality rates, with 34% of patients succumbing within 30 days. This study aimed to develop predictive models for VISA (including *h*VISA) bacteremia outcomes using data mining techniques, potentially improving patient management and therapy selection. We focused on three endpoints in patients receiving traditional vancomycin therapy: VISA persistence in bacteremia after 7 days, after 30 days, and patient mortality. Our analysis incorporated 29 risk factors associated with VISA bacteremia. The resulting models demonstrated high predictive accuracy, with 82.0–86.6% accuracy for 7-day VISA persistence in blood cultures and 53.4–69.2% accuracy for 30-day mortality. These findings suggest that data mining techniques can effectively predict VISA bacteremia outcomes in clinical settings. The predictive models developed have the potential to be applied prospectively in hospital settings, aiding in risk stratification and informing treatment decisions. Further validation through prospective studies is warranted to confirm the clinical utility of these predictive tools in managing VISA infections.

## 1. Introduction

Vancomycin-intermediate *Staphylococcus aureus* (VISA) bacteremia has emerged as a significant concern in Intensive Care Units (ICUs) worldwide. The prevalence of VISA infections in ICUs has been steadily increasing, with recent studies reporting rates ranging from 2% to 10% among methicillin-resistant *Staphylococcus aureus* (MRSA) isolates [1,2,3]. This rise is particularly alarming given the limited treatment options available for VISA infections and the high mortality rates associated with VISA bacteremia, which can exceed 30% within 30 days of infection onset [4,5]. Despite rigorous infection control measures, the persistence of VISA in clinical settings underscores the urgent need for improved strategies in detecting, treating, and managing these infections [6,7,8].

Numerous studies have found that a high vancomycin MIC is associated with higher mortality and treatment failure [9]. A critical challenge in managing VISA bacteremia is the limited antibiotic options for treatment. The reduced efficacy of vancomycin requires using alternative antibiotics like linezolid, daptomycin, and tigecycline. However, access to and familiarity with these newer antibiotics varies [10]. Furthermore, traditional microbiological methods for detecting reduced vancomycin susceptibility are time-consuming, often requiring 24 to 48 h for results, and can lead to inadequate antibiotic therapy, potentially resulting in treatment failure, prolonged hospital stays, and increased mortality [11,12]. Moreover, the subtle phenotypic changes associated with VISA can sometimes lead to misclassification, further complicating timely and accurate diagnosis [13]. Even a small increase in the MIC below the susceptibility breakpoint can affect the clinical efficacy of glycopeptides. Thus, treating VISA among MRSA patients is complex [14]. Therefore, existing clinical and laboratory diagnostic methods may be inadequate due to the delay in determining the most appropriate treatment strategy. Early predictive modeling based on large-scale clinical data identifies resistance patterns and predicts antimicrobial resistance development [15].

Recent advances in machine learning (ML) for healthcare analytics have greatly enhanced predictive modeling, particularly in identifying antimicrobial resistance patterns through genomic data analysis and laboratory results [15,16]. These technologies facilitate faster and more appropriate diagnostics, enabling healthcare providers to optimize antibiotic use and improve patient outcomes. Despite challenges such as data quality and model validation, integrating ML into clinical practice may hold promise for transforming healthcare delivery. Data mining techniques may solve this problem by leveraging clinical data to predict VISA infections before traditional diagnostic methods can confirm them [17]. Early identification of potential VISA cases could enable clinicians to promptly initiate more appropriate antibiotic regimens, such as daptomycin or linezolid, instead of relying solely on vancomycin [18]. This proactive approach may significantly improve patient outcomes and reduce the spread of resistant organisms within healthcare settings [19]. Therefore, with data mining and artificial intelligence, software applications based on patient information may assist clinicians in prescribing appropriate antimicrobial therapy more effectively for the treatment of MRSA bloodstream infections [16,19,20,21,22]

Thus, the primary objective of this study is to develop and validate a predictive model using data mining techniques to identify patients at high risk of VISA bacteremia in ICU settings. By analyzing a comprehensive set of clinical, laboratory, and demographic variables, we aim to create a tool that can accurately predict VISA infections earlier than conventional methods. Additionally, this study seeks to identify key risk factors associated with VISA bacteremia persistence and mortality [12]. This study’s outcome may provide clinicians with a valuable decision-support tool for early VISA detection, inform empiric antibiotic selection in high-risk patients, and ultimately improve patient outcomes. Furthermore, elucidating important predictors of VISA infections, this study may guide future research into targeted prevention strategies and novel therapeutic approaches for managing these challenging infections [23].

## 2. Methods

### 2.1. Definition of Vancomycin MIC and hVISA/VISA Identification

The study hospital was a medical center located in southern Taiwan. This data analysis received approval from the Institutional Ethics Review Board during the clinical investigations (E-Da Hospital, EMRP-098-042) [4]. The study enrolled 284 consecutive patients with MRSA bacteremia who received glycopeptide therapy after the initial blood culture identified MRSA. Vancomycin minimum inhibitory concentrations (MICs) were determined using the Etest method (AB BIODISK, Solna, Sweden). MIC testing was performed without knowledge of clinical outcomes to ensure unbiased results. The study categorized isolates into two groups based on their vancomycin MICs: the “high MIC” group included isolates with an Etest MIC ≥ 1.5 mg/L, while the “low MIC” group comprised isolates with MICs of 1 mg/L or 0.5 mg/L. The area under the curve (AUC) was calculated for each test strain to determine the ratio of AUCtest/AUCMu3 (Mu3 is a known *h*VISA control strain) [24]. The criteria for classification were (1) VSSA (vancomycin-susceptible *S. aureus*): AUCtest/AUCMu3 ratio < 0.9; (2) *h*VISA: AUCtest/AUCMu3 ratio between 0.9 and 1.3; (3) VISA: AUCtest/AUCMu3 ratio > 1.3.

### 2.2. Data Collection

We collected the demographic and clinical characteristics of each study patient and the MICs and VISA statuses in MRSA isolates. A total of 29 risk factors were collected, including the underlying comorbidities and infection syndromes, identified based on clinical, bacteriological, and radiological investigations defined by USCDC and others (Table 1) [25,26,27]. We defined persistent bloodstream infection as a breakthrough bloodstream infection occurring after 7 days of vancomycin treatment.

### 2.3. Data Mining Methodology

Binary classification, a foundation of machine learning and statistical analysis, was used in this study for predicting outcomes with two possible classes, such as the presence or absence of VISA infections. Various methods have been developed to address this problem, each with unique strengths and characteristics. Neural Networks, inspired by biological neural systems, consist of interconnected nodes organized in layers [28]. They excelled at capturing complex non-linear relationships in data, making them powerful for binary classification tasks. Decision tree-based methods, including Classification and Regression Trees (C&R Tree) [29], Quick, Unbiased, Efficient Statistical Tree (QUEST) [30], Chi-squared Automatic Interaction Detection (CHAID) [31], and C5.0 [32], offer interpretable models by recursively partitioning data based on feature values. C&R Trees use the Gini index or information gain for splitting, while QUEST employs statistical tests to select variables. CHAID utilizes chi-square tests for categorical predictors and F-tests for continuous ones, and C5.0 is an improved version of the C4.5 algorithm, offering boosting capabilities. Logistic Regression, a statistical method, models the probability of the binary outcome using a logistic function of the input features, providing easily interpretable coefficients. Bayesian Networks (Bayes Net), introduced by Pearl [33], use probabilistic graphical models to represent relationships between variables and perform classification based on conditional probabilities. Lastly, Support Vector Machines (SVM), developed by Cortes and Vapnik in 1995, aim to find the hyperplane that best separates the two classes in a high-dimensional space, often using kernel functions to handle non-linear relationships [34].

Each of these methods has its advantages and limitations. Neural Networks and SVMs often perform well with large, complex datasets but may require more computational resources and be less interpretable. Decision tree-based methods and Decision Lists offer clear insights into the decision-making process but can be prone to overfitting [32]. Logistic Regression and Discriminant Analysis provide easily interpretable results and work well when the relationship between features and the outcome is approximately linear [35]. Bayes Net is particularly useful when prior knowledge about the problem domain is available [36]. Nearest Neighbor methods are simple to implement and can capture complex decision boundaries but may struggle with high-dimensional data [37].

The method choice depends on the dataset’s specific characteristics, the desired balance between model performance and interpretability, and computational constraints. In practice, it is often beneficial to compare the performance of multiple methods using appropriate evaluation metrics such as accuracy, precision, recall, F1-score, and area under the ROC curve [38] to determine the most suitable approach for predicting visa infections. Recent comparative studies have shown that ensemble methods and advanced techniques like gradient boosting often outperform individual classifiers, highlighting the importance of considering a wide range of approaches when tackling binary classification problems [39].

IBM SPSS Modeler (v18.2, IBM Corp., Armonk, NY, USA) was used to build the prediction model. The auto classifier node supports model types such as Neural Net, C&R Tree, QUEST, CHAID, C5.0, and Logistic Regression. Decision List, Bayes Net, Discriminant, Nearest Neighbor, and Support Vector Machine (SVM) were used to compare the model performance of binary (Yes/No) data.

### 2.4. Risk Factors

Based on the literature, which indicates the risk factors associated with MRSA, the investigators selected 29 predictive factors (Table 1) from patients’ electronic charts [26,27].

### 2.5. Training Model

To evaluate the accuracy of our model, we divided the patient data into two datasets: 50% for training and 50% for model validation. The software randomly selected 50% of records from the data pool for training and used the remaining 50% for validation. This research used the software’s built-in balance node for positive and negative data balance. This balance node worked by duplicating or discarding records in the dataset based on the balancing directives specified in the node [40]. A factor greater than one results in records being duplicated in the dataset, while a factor lower than one results in records being discarded. The records for duplication and discarding are randomly selected for non-integer factors. We assigned the node option to “only balance training data”, which is selected by default. Therefore, the results may differ slightly from one execution to the next. The software’s default settings rejected the models with prediction accuracy lower than 50% (no better than Toss a Coin).

## 3. Results

In this study, we analyzed 284 patients with MRSA bloodstream infections, of which 16 were confirmed as VISA using a molecular method (multilocus sequence typing and pulsed-field gel electrophoresis of XbaI-digested genomic DNA) [4]. Ten patients had an E Test result of >3.0 on vancomycin, and 174 patients had an E Test result of 2.0 on vancomycin. Of the 284 patients, 46 had persistence of *S. aureus* in blood cultures after 7 days, 25 had persistence after 30 days, and 104 patients died within 30 days of ICU stay.

We identified 29 clinical signs as risk factors (Xs) and used the persistence of *S. aureus* in blood cultures after 7 days (Y1), 30 days (Y2), and death (Y3) as our endpoints (Ys). We first applied the Binary Classifier analysis to the 29 risk factors, which suggested appropriate models and average prediction accuracy rates (listed in Table 2).

Four models were applicable for predicting the seven-day persistence of *S. aureus* in blood cultures. The C&R Tree model had an accuracy of 86.6%, with 25% false positives and 12.1% false negatives. The Neural Net model had an accuracy of 82.0%, with 72.73% false positives and 15.8% false negatives. The QUEST 1 model had an accuracy of 83.8%, with 16.2% false negatives. The C 5.1 model had an accuracy of 85.2%, with 0% false positives and 15% false negatives (Table 3). The key risk factors used in the four models ranged from 2 to 15, with “Endocarditis with Intracardiac catheter” appearing in all four models (Table 4).

Only one model, the Neural Network, was applicable for predicting the 30-day persistence of *S. aureus*, while C&R Tree, QUEST 1, and C 5.1 have prediction accuracy lower than 50%. The prediction accuracy was 91.2%, with 8.8% false negatives (Table 5). Fourteen key factors were recorded (Table 6).

Three models were applicable for predicting death. The Neural Net model had an accuracy of 65.1%, with 46.6% false positives and 30.8% false negatives. The C&R Tree model had an accuracy of 72.5%, with 26.8% false positives and 27.6% false negatives. The C 5.1 model had an accuracy of 72.2%, with 23.4% false positives and 28.7% false negatives (Table 7). The key risk factors ranged from 8 to 14, with “End organ damage due to diabetes” and “Surgery” being two common factors among these three models (Table 8).

**Figure 1:**
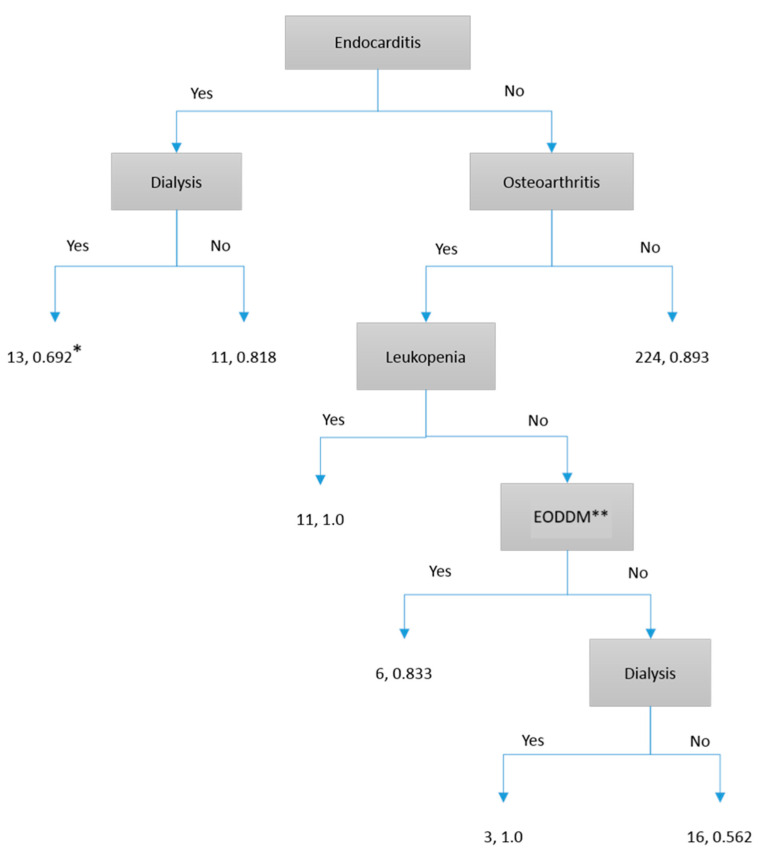
C&R Tree model prediction for 7 days persistence of *S. aureus* in blood cultures. * The first number represents the number of patients, and the second number represents prediction accuracy. ** EODDM: End organ damage of diabetes.

**Figure 2:**
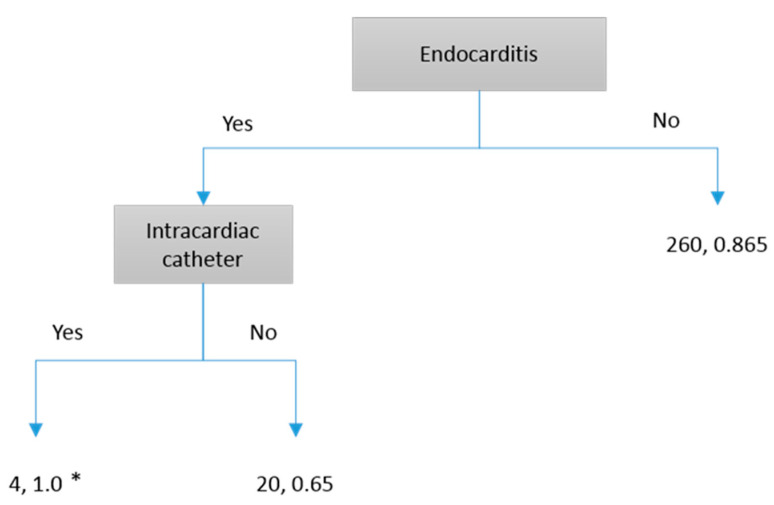
C 5.1 Model prediction for 7 days persistence of *S. aureus* in blood cultures. * The first number represents the number of patients, and the second number represents prediction accuracy.

**Figure 3:**
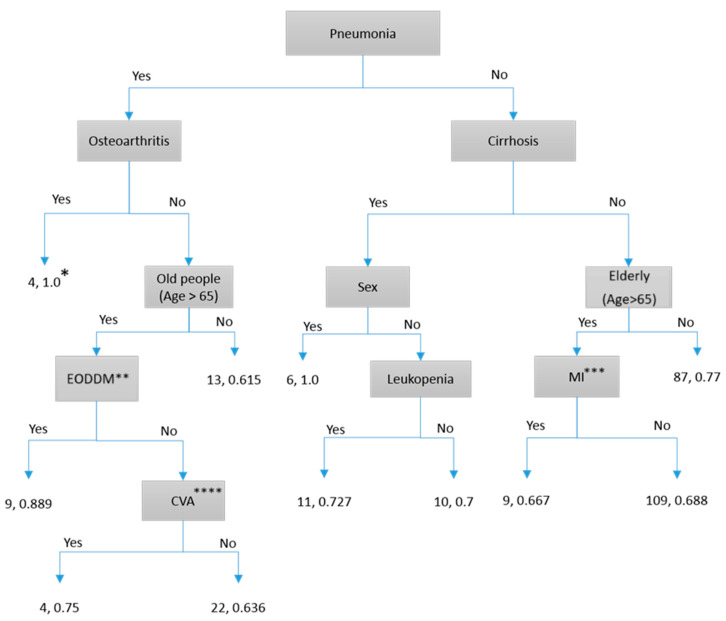
C&R Tree model prediction of death within 30 days. * The first number represents the number of patients, and the second number represents prediction accuracy. ** EODDM: End organ damage of diabetes. *** MI: Myocardial infarct. **** Cerebral vascular disease.

**Figure 4:**
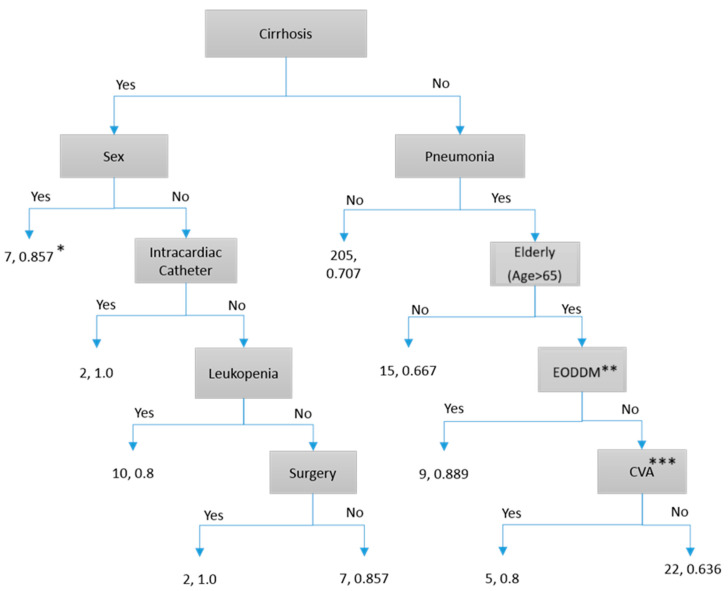
C 5.1 model prediction of death within 30 days. * The first number represents the number of patients, and the second number represents prediction accuracy. ** EODDM: End organ damage of diabetes. *** Cerebral vascular disease.

To facilitate the application of our study results in future clinical practice, we found that some models required many risk factors and were challenging to implement (e.g., Neural Network). For clinicians’ daily use, a simple decision tree with Yes/No routes would be the most practical way to use our prediction results. We found that four decision-tree models can be used to predict patient outcomes:

1. Predicting the 7-day persistence of *S. aureus* bloodstream infection via the C&R Tree model: As shown in Figure 1, “Endocarditis” is the first determining factor. Following the routes, we can obtain 56.2~100% accuracy.
2. Predicting the 7-day persistence of *S. aureus* by the C 5.1 model: As shown in Figure 2, “Endocarditis” is the first determining factor. Following the routes, we can obtain accuracy between 65% and 100%.
3. Predicting death due to *S. aureus* by the C&R Tree model: As shown in Figure 3, “Pneumonia” is the first determining factor. Following the routes, we can obtain 61.5~100% accuracy.
4. Predicting death due to *S. aureus* by the C 5.1 model: As shown in Figure 4, “Cirrhosis” is the first determining factor. Following the routes, we can obtain 63.6~100% accuracy.

## 4. Discussion

In recent years, there has been a noted increase in the minimum inhibitory concentrations (MICs) of vancomycin among MRSA isolates in the bloodstream [41,42]. Even minor increases in the MIC below the susceptibility breakpoint can impact the clinical effectiveness of glycopeptides. When MRSA is confirmed via laboratory diagnosis, not switching to an effective antimicrobial is linked to increased morbidity and mortality [43,44]. During the recent pandemic, COVID-19 patients who developed MRSA bloodstream infections showed significantly higher mortality rates compared to patients with either pathogen alone. The rate of healthcare facility-onset MRSA bacteremia was approximately 5 times higher in COVID-19 patients compared to non-COVID-19 patients, with studies showing that SARS-CoV-2 infection makes patients more susceptible to MRSA coinfections. These findings suggest that multiple factors contributed to this increased risk, including mechanical ventilation, central lines, corticosteroids, and increased antibiotic use (particularly ceftriaxone) during the pandemic [45,46].

Our study found that out of 16 patients confirmed with VISA, 6 did not show persistence of *S. aureus* in blood cultures 7-day and 30-day post-initiation of vancomycin treatment. If the laboratory diagnosis of VISA is a predictor of standard vancomycin treatment failure, the accuracy is only 62.5%. In contrast, among 173 patients with a vancomycin MIC of 2 µg/mL, where vancomycin should be effective against the infecting *S. aureus*, 31 patients showed persistence of *S. aureus* after 7 days, and 14 patients showed persistence after 30 days. The accuracies of predicting the 7-day and 30-day persistence of *S. aureus* using laboratory MIC data are only 82.1% and 91.9%, respectively. Although laboratory diagnosis is considered an evidence-based method, it does not achieve 100% accuracy [44].

We attempted to predict the outcomes of patients with MRSA bloodstream infection using three indicators: 7-day persistence, 30-day persistence, and death. The risk factors used for input are not laboratory-related and can be readily available during antibiotics administration. Based on our results, we can predict the 7-day persistence of MRSA in blood cultures with an accuracy ranging from 82.0% to 86.6%, 30-day persistence of MRSA in blood cultures with an accuracy of 91.2%, and patient death within 30 days with an accuracy ranging from 53.4% to 69.2%. Although our results may be similar to laboratory diagnosis, our method is cost-effective. We also propose decision tree algorithms that are easy for clinicians to use when making calculations or predictions. However, the prediction of 30-day persistently positive bacteremia patients has a rate of 91.2%. Still, since late bacteremia patients may have persistent infections due to other complications and a high risk of acquiring nosocomial infections, VISA may not be a determining factor, and the prediction accuracy was suboptimal [44].

The following important question is, assuming our prediction is valid, what can or what will clinicians do if they know that a particular patient will likely have MRSA persistence in the blood cultures 7 days or 30 days after the standard vancomycin treatment1? How would a clinician use our prediction to improve the efficacy of antibiotic treatment? Currently, there may be a few options available:

(1) Use a higher dose, longer course regimen of vancomycin: High doses of vancomycin may result in better efficacy. A prospective study in patients with staphylococcal lower respiratory tract infections showed that vancomycin AUC/MIC ratios of >400 gave much better clinical outcomes (78%) than ratios below that ratio (23%) [44,47,48,49].
(2) Other antibiotics than vancomycin: The optimum treatment for vancomycin-intermediate *S. aureus* has yet to be established. Combining ceftaroline/ceftobiprole and daptomycin has demonstrated efficacy in treating persistent bacteremia, including cases involving VISA isolates [50,51,52,53]. In Japan, most experience has been gained with the combinations of agents such as ampicillin–sulbactam and arbekacin, an aminoglycoside approved specifically for MRSA treatment in Japan [54]. Other options include quinupristin–dalfopristin and linezolid. Some animal models experience support using other beta-lactam with vancomycin, such as ampicillin–sulbactam [55] and nafcillin [56]. Depending on their availability, other agents used over the years for treating severe MRSA infections might also be considered, such as fosfomycin with or without beta-lactam [44,57,58,59].

The strength of our approach lies in its ability to utilize readily available clinical information, avoiding the delays associated with traditional laboratory-based diagnostics. This timeliness could prove crucial in the early stages of treatment, potentially allowing for more rapid and targeted interventions. Moreover, the decision tree algorithms developed offer clinicians a user-friendly tool, facilitating quick bedside risk assessments. However, it is important to note our study’s limitations. The retrospective nature of the imbalanced data and the single-center design (without external validation data) may limit generalizability. Additionally, while our models show promising accuracy, they are not infallible, and clinical judgment remains paramount in patient care decisions. Other challenges associated with AI use in healthcare include reproducibility issues and the need for robust evidentiary standards to ensure reliable outcomes. Trust issues arise from algorithmic biases and a lack of transparency, undermining confidence among healthcare practitioners and patients. Additionally, ethical, legal, and societal concerns, such as data privacy and the equitable distribution of AI benefits, complicate the adoption process. At the same time, post-adoption uncertainty may lead to variability in AI performance and clinical decision-making [60]. Thus, we call for more clinicians to participate in such studies with valuable data, and a systematic feature selection from the patient data from the electronic chart system may improve the performance and robustness of the algorithms. Furthermore, this study did not incorporate MIC values (EUCAST and BSAC: MIC ≤ 2 mg/L—sensitive, >2 mg/L—resistant; CLSI: MIC ≤ 2 mg/L—sensitive, 4–8 mg/L—intermediate (VISA), ≥16 mg/L—resistant (VRSA)) in the prediction models. The nature of false positive/negative was not discussed, which was also a notable limitation.

The implications of this research extend beyond immediate clinical applications. Our findings highlight several key areas for future investigation and potential improvements in MRSA and VISA management:

(1) Personalized Treatment Strategies: The ability to predict treatment outcomes opens the door to more personalized antibiotic regimens. For patients identified as high risk for persistent bacteremia or VISA, clinicians could consider alternative strategies such as higher vancomycin dosing, extended treatment durations, or an early switch to alternative antibiotics like daptomycin or linezolid.
(2) Antibiotic Stewardship: By identifying patients at lower risk for complications, our model could support more judicious use of broad-spectrum antibiotics, potentially reducing the selective pressure for antibiotic resistance.
(3) Clinical Trial Design: Pharmaceutical companies could utilize similar predictive models to stratify patients in clinical trials, potentially leading to more targeted and efficient evaluations of new antibiotics for MRSA and VISA infections.
(4) Real-time Decision Support: Future iterations of this model could be integrated into electronic health record systems, providing clinicians with real-time risk assessments and treatment recommendations.
(5) Continuous Learning Models: As suggested above, there is potential to develop continuously updating predictive models that incorporate new patient data in real-time, constantly refining their accuracy and adapting to evolving bacterial resistance patterns.
(6) Cost-effectiveness: While not directly assessed in this study, the potential for early identification of high-risk patients could lead to more efficient resource allocation and potentially reduce overall healthcare costs associated with prolonged treatments and complications.
(7) Interdisciplinary Collaboration: This work underscores the value of collaboration between data scientists, infectious disease specialists, and clinical microbiologists in addressing complex healthcare challenges.

## 5. Conclusions

This study demonstrates the potential of data mining techniques to predict outcomes in patients with methicillin-resistant *Staphylococcus aureus* (MRSA) bacteremia, particularly in identifying cases that may progress to vancomycin-intermediate *Staphylococcus aureus* (VISA) infections. By analyzing 29 risk factors, we developed models capable of predicting the persistence of *S. aureus* in blood cultures after 7 and 30 days of vancomycin treatment, as well as 30-day mortality rates. Our findings reveal impressive predictive accuracies: 82.0% to 86.6% for 7-day persistence, 91.2% for 30-day persistence, and 53.4% to 69.2% for 30-day mortality. These results underscore the potential of data mining approaches to enhance clinical decision-making in managing MRSA bacteremia and potential VISA cases. Furthermore, this study represents a significant step forward in applying data mining techniques to the challenging realm of antibiotic-resistant infections. By harnessing the power of readily available clinical data, we have developed a promising tool for predicting outcomes in MRSA bacteremia and potential VISA cases. While further validation and refinement are necessary, these findings lay the groundwork for a more proactive, data-driven approach to managing these serious infections. As we continue to face the growing threat of antibiotic resistance, innovative approaches combining clinical expertise with advanced data analysis techniques will be crucial in our ongoing efforts to improve patient outcomes and combat the spread of resistant pathogens.

## Figures and Tables

**Table 1:** Risk factors used in predictive model construction.

1	Age	16	Hepatitis
2	AIDS	17	Leukemia
3	Autoimmune	18	Leukopenia
4	Blood culture collection at the emergency room	19	Lymphoma
5	Osteomyelitis	20	Metastatic Cancer
6	Intracardiac catheter	21	Myocardial infarct
7	Cerebral vascular disease (CVA)	22	Elderly (Age > 65)
8	Chronic obstructive pulmonary disease (COPD)	23	Peptic ulcer disease (pud)
9	Cirrhosis	24	Peripheral arterial occlusive disease (POAD)
10	Congestive heart failure (CHF)	25	Pneumonia
11	Diabetes	26	With prosthetic joint
12	Dialysis	27	Sex
13	End organ damage of diabetes (EODDM)	28	Surgery
14	Endocarditis	29	White blood count (WBC)
15	Hemiparesis		

**Table 2:** Results of predictive models using 29 risk factors.

Predictive Model	7 Days Persistence			30 Days Persistence	Death		
	C&R Tree	Neural Net	QUEST 1	Neural Net	Neural Net	C&R Tree	C 5.1
1	86.62	86.620	83.80	91.197	63.380	72.535	72.183
2	86.62	84.155	83.80	91.197	64.789	72.535	72.183
3	86.62	85.915	83.80	91.197	65.845	72.535	72.183
4	86.62	83.800	83.80	91.197	63.380	72.535	72.183
5	86.62	83.800	83.80	91.197	66.197	72.535	72.183
6	86.62	81.690	83.80	91.197	60.563	72.535	72.183
7	86.62	83.800	83.80	91.197	64.789	72.535	72.183
8	86.62	84.859	83.80	91.197	66.197	72.535	72.183
9	86.62	83.800	83.80	91.197	64.437	72.535	72.183
10	86.62	85.211	83.80	91.197	65.141	72.535	72.183
Average Accuracy (%)	86.6	84.4	83.8	91.2	64.5	72.5	72.2

**Table 3:** Results of prediction in 7 days persistence of *S. aureus* in blood cultures.

Model		C&R Tree		Neural Net		QUEST 1		C 5.1	
Overall Results	Correct	246	86.62%	233	82.04%	238	83.80%	242	85.21%
	Incorrect	38	13.80%	51	17.92%	46	16.2%	42	14.79%
	Total	284		284		284		284	
Type 1 error	Correct	12	75.00%	3	27.27%			4	100.00%
	Incorrect	4	25.00%	8	72.73%			0	0.00%
	Total	16		11				4	
Type 2 error	Correct	234	87.31%	230	84.25%	238	83.80%	238	85.00%
	Incorrect	34	12.69%	43	15.75%	46	16.2%	42	15.00%
	Total	268		273		284		280	

**Table 4:** Risk factors used in prediction for 7 days persistence of *S. aureus* in blood cultures.

Model	C&R Tree (*n* = 14)	Neural Net (*n* = 15)	QUEST 1 (*n* = 0)	C 5.1 (*n* = 2)
1. Age	√			
2. AIDS	√			
3. Autoimmune	√	√		
4. Blood culture collection at the emergency room				
5. Osteomyelitis	√	√		
6. Intracardiac catheter	√	√		√
7. Cerebral vascular disease (CVA)		√		
8. Chronic obstructive pulmonary disease (COPD)				
9. Cirrhosis	√	√		
10. Congestive heart failure (CHF)				
11. Diabetes	√	√		
12. Dialysis	√			
13. End organ damage of diabetes (EODDM)	√			
14. Endocarditis	√	√		√
15. Hemiparesis		√		
16. Hepatitis				
17. Leukemia				
18. Leukopenia	√	√		
19. Lymphoma		√		
20. Metastatic Cancer		√		
21. Myocardial infarct		√		
22. Elderly (Age > 65)	√	√		
23. Peptic ulcer disease (pud)		√		
24. Peripheral arterial occlusive disease (POAD)	√			
25. Pneumonia				
26. With prosthetic joint				
27. Sex		√		
28. Surgery	√			
29. White blood count (WBC)				

“√” represents such factors were used in the prediction models.

**Table 5:** Results of prediction in 30 days persistence of *S. aureus* in blood cultures.

Model		Neural Net	
Overall Results	Correct	259	91.20%
	Incorrect	25	8.80%
	Total	284	
Type 2 error	Correct	259	91.20%
	Incorrect	25	8.80%
	Total	284	

**Table 6:** Risk factors used in prediction for 30 days persistence of *S. aureus* in blood cultures.

Model	Neural Net (*n* = 14)
1. Age	
2. AIDS	√
3. Autoimmune	√
4. Blood culture collection at the emergency room	
5. Osteomyelitis	√
6. Intracardiac catheter	√
7. Cerebral vascular disease (CVA)	√
8. Chronic obstructive pulmonary disease (COPD)	
9. Cirrhosis	
10. Congestive heart failure (CHF)	
11. Diabetes	
12. Dialysis	
13. End organ damage of diabetes (EODDM)	
14. Endocarditis	
15. Hemiparesis	√
16. Hepatitis	√
17. Leukemia	√
18. Leukopenia	
19. Lymphoma	√
20. Metastatic Cancer	√
21. Myocardial infarct	
22. Elderly (Age > 65)	
23. Peptic ulcer disease (pud)	√
24. Peripheral arterial occlusive disease (POAD)	√
25. Pneumonia	√
26. With prosthetic joint	
27. Sex	√
28. Surgery	
29. White blood count (WBC)	

“√” represents such factors were used in the prediction models.

**Table 7:** Results of prediction in death within 30 days.

Model		Neural Net		C&R Tree		C 5.1	
Overall Results	Correct	185	65.14%	206	72.54%	205	72.18%
	Incorrect	99	34.86%	78	27.46%	79	27.82%
	Total	284		284		284	
Type 1 error	Correct	39	53.42%	41	73.21%	36	76.60%
	Incorrect	34	46.58%	15	26.79%	11	23.40%
	Total	73		56		47	
Type 2 error	Correct	146	69.19%	165	72.37%	169	71.31%
	Incorrect	65	30.81%	63	27.63%	68	28.69%
	Total	211		228		237	

**Table 8:** Risk factors used in the prediction of death.

Model	Neural Net (*n* = 14)	C&R Tree (*n* = 8)	C 5.1 (*n* = 9)
1. Age	√		
2. AIDS	√		
3. Autoimmune	√		
4. Blood culture collection at the emergency room	√		
5. Osteomyelitis	√		
6. Intracardiac catheter	√		√
7. Cerebral vascular disease (CVA)	√		√
8. Chronic obstructive pulmonary disease (COPD)			
9. Cirrhosis			√
10. Congestive heart failure (CHF)	√		
11. Diabetes			
12. Dialysis	√		
13. End organ damage of diabetes (EODDM)	√	√	√
14. Endocarditis		√	
15. Hemiparesis		√	
16. Hepatitis		√	
17. Leukemia	√		
18. Leukopenia		√	√
19. Lymphoma	√		
20. Metastatic Cancer			
21. Myocardial infarct			
22. Elderly (Age > 65)			√
23. Peptic ulcer disease (pud)	√		
24. Peripheral arterial occlusive disease (POAD)		√	
25. Pneumonia		√	√
26. With prosthetic joint			
27. Sex			√
28. Surgery	√	√	√
29. White blood count (WBC)			

“√” represents such factors were used in the prediction models.

## Data Availability

The data presented in this study are available on request from the corresponding author. The data are not publicly available due to inappropriate commercial uses.
